# Supplementary material for: Signatures of Cholera Outbreak during the Yemeni Civil War, 2016–2019
Source: Int J Environ Res Public Health. 2021 Dec 30;19(1):378. doi: 10.3390/ijerph19010378 (PMC8744546; doi:10.3390/ijerph19010378)
Supplement: Supplementary file 1 [file ijerph-19-00378-s001.zip › Yemen_KZFilter_Supplementary_20211229.pdf]

Supplementary Material

Signatures of cholera outbreak during the Yemeni Civil War, 2016-2019

Ryan B. Simpson [1,*], Sofia Babool [2], Maia C. Tarnas [3], Paulina M. Kaminski [1], Meghan A. Hartwick [1], and Elena N. Naumova [1,*]

[1] Department of Nutrition Epidemiology and Data Science, Tufts University Friedman School of Nutrition Science and Policy, 150 Harrison Avenue, Boston, MA 02111, USA; ryan.simpson@tufts.edu; paulina.kaminski13@gmail.com; meghartwick@gmail.com; elena.naumova@tufts.edu

[2] Department of Neuroscience, The University of Texas at Dallas, 800 W Campbell Road, Richardson, TX 75080, USA; sfb170000@utdallas.edu

[3] Department of Community Health, Tufts University School of Arts and Sciences, 574 Boston Avenue, Medford, MA 02155, USA; maia.tarnas@gmail.com

* Correspondence: ryan.simpson@tufts.edu; Tel.: 978-697-1037
elena.naumova@tufts.edu; Tel.: 617-636-2927

Supplementary Excel File. This file provides Excel Tables S1-S5. Excel Table S1 includes weekly time series data of laboratory-confirmed cholera cases and rates (cases per 100,000 persons) for 20 of 21 governorates in Yemen (excluding the Hadramaut and Socotra island governorate where data were not consistently reported) from Week 36 of 2016 (04-September) through Week 52 of 2019 (29-December). We extracted, harmonized, and integrated data from daily, weekly, and monthly epidemiological bulletins reported by the World Health Organization's (WHO) Eastern Mediterranean Regional Office (EMRO) [4,28-30,42,43]. The final weekly time series dataset provides cholera cases and rates with the presence of missing data and linear approximation imputations for estimating missing records. The remaining Excel Tables S2-S5 provide the curated dataset used for all analyses of this research as well as subsets of this dataset to create targeted multi-panel, shared-axis data visualizations. We calculated cholera rates using weekly pro-rated population estimates that adjusted for conflict fatalities and a low-to-moderate population growth rate. First, we calculated the population for Week 1 of 2017 as the average of multiple sources including the WHO EMRO, the 2004 Yemeni Central Statistical Organization (CSO) population projection for 2005-2025, and the International Organization for Migration (IOM) Displacement Tracking Matrix's (DTM) 2017 Population Estimate [30,35,36]. Next, we estimated weekly governorate-level population forwards from Week 2 of 2017 through Week 52 of 2019 by subtracting conflict fatalities and multiplying the difference by the prorated annual growth rate. We estimated weekly population backwards from Week 52 of 2016 through Week 36 of 2016 by adding conflict fatalities and dividing the sum by the weekly-adjusted population growth rate. We estimated smoothed rates (laboratory-confirmed cholera cases per 100,000 persons) and trivial derivative values (Δ rates per epidemiological week) using Kolmogorov Zurbenko (KZ) adaptive filters. We selected the best performing smoother ($Z^*_{t,s,3-5}$) as the average of a 3-week ($Z^*_{t+1,s,3}$) and 5-week ($Z^*_{t+1,s,5}$) window size and trivial derivative as the absolute change in weekly smoothed rates calculated as: $Z'_{t,s,3-5} = \left(Z^*_{t+1,s,3} + Z^*_{t+1,s,5}\right)/2 - \left(Z^*_{t,s,3} + Z^*_{t,s,5}\right)/2$. We also provide links to subnational shapefiles for developing cluster maps and all R codes used to develop data visualizations found within the main text of this manuscript [41].

Supplementary Table S1. Weak serial synchronization of confirmed cholera rates between 20 Yemeni governorates (excludes the Hadramaut and Socotra island governorate) from Week 36 of 2016 (04-11-September) through Week 52 of 2019 (23-29-December).

Governorate	Governorate	+6	+5	+4	+3	+2	+1	0	-1	-2	-3	-4	-5	-6
Sana'a	Sana'a City	0.663	0.711	0.756	0.797	0.833	0.860	0.874	0.880	0.873	0.859	0.841	0.819	0.791
Sana'a	Al-Hudaydah	0.830	0.849	0.861	0.863	0.856	0.841	0.817	0.787	0.750	0.708	0.665	0.625	0.589
Sana'a City	Al-Hudaydah	0.892	0.896	0.889	0.872	0.850	0.823	0.790	0.754	0.712	0.665	0.618	0.572	0.527
Amran	Al-Mahwit	0.803	0.837	0.868	0.894	0.915	0.926	0.925	0.910	0.884	0.847	0.802	0.752	0.699
Amran	Dhamar	0.884	0.915	0.940	0.956	0.962	0.957	0.940	0.911	0.873	0.829	0.780	0.727	0.672
Amran	Al-Bayda	0.623	0.643	0.663	0.680	0.694	0.700	0.698	0.697	0.688	0.672	0.649	0.620	0.586
Al-Mahwit	Dhamar	0.845	0.884	0.917	0.942	0.956	0.960	0.952	0.932	0.903	0.867	0.825	0.780	0.732
Al-Mahwit	Al-Bayda	0.685	0.716	0.747	0.777	0.802	0.820	0.826	0.827	0.818	0.801	0.779	0.752	0.720
Dhamar	Al-Bayda	0.615	0.651	0.687	0.722	0.753	0.777	0.794	0.810	0.817	0.814	0.802	0.782	0.753
Raymah	Ibb	0.669	0.712	0.753	0.790	0.820	0.838	0.842	0.835	0.820	0.800	0.778	0.756	0.733
Raymah	Taizz	0.727	0.768	0.805	0.836	0.859	0.874	0.878	0.875	0.866	0.851	0.833	0.813	0.791
Ibb	Taizz	0.808	0.844	0.876	0.903	0.923	0.935	0.937	0.927	0.905	0.875	0.839	0.800	0.757
Al-Jawf	Hajjah	0.837	0.868	0.896	0.920	0.940	0.952	0.957	0.955	0.946	0.933	0.917	0.898	0.876
Al-Jawf	Sa'ada	0.774	0.789	0.803	0.814	0.823	0.828	0.825	0.817	0.804	0.787	0.768	0.750	0.731
Hajjah	Sa'ada	0.729	0.742	0.753	0.762	0.768	0.771	0.767	0.759	0.745	0.727	0.707	0.687	0.666
Al-Dhale'e	Abyan	0.718	0.741	0.759	0.772	0.778	0.775	0.766	0.750	0.727	0.699	0.665	0.624	0.580
Al-Dhale'e	Aden	0.740	0.756	0.765	0.767	0.763	0.751	0.735	0.716	0.694	0.669	0.636	0.598	0.555
Al-Dhale'e	Lahj	0.743	0.759	0.772	0.783	0.790	0.793	0.787	0.771	0.748	0.719	0.687	0.652	0.616
Abyan	Aden	0.764	0.790	0.812	0.830	0.842	0.848	0.846	0.843	0.830	0.809	0.781	0.747	0.709
Abyan	Lahj	0.743	0.768	0.788	0.804	0.817	0.826	0.827	0.822	0.809	0.789	0.765	0.739	0.713
Aden	Lahj	0.773	0.812	0.843	0.864	0.874	0.877	0.871	0.859	0.839	0.814	0.785	0.753	0.718
Marib	Al-Maharah	0.546	0.602	0.651	0.693	0.726	0.749	0.764	0.767	0.765	0.761	0.748	0.730	0.710
Marib	Shabwah	0.674	0.716	0.754	0.788	0.814	0.834	0.843	0.843	0.836	0.826	0.816	0.805	0.795
Al-Maharah	Shabwah	0.713	0.752	0.792	0.829	0.859	0.880	0.888	0.876	0.855	0.829	0.801	0.766	0.730

We assessed serial synchronization using Spearman cross correlations from -6 to +6 lags. We report correlations coefficients in the table above and note that all coefficients are significant at $\alpha < 0.001$. Correlation estimates were performed using smoothed rates (laboratory-confirmed cholera cases per 100,000 persons) produced with Kolmogorov Zurbenko (KZ) adaptive filters. We selected the best performing smoother ($Z^*_{t,s,3-5}$) as the average of a 3-week ($Z^*_{t+1,s,3}$) and 5-week ($Z^*_{t+1,s,5}$) window size. We estimated smoothed rates for each governorate using 172 weeks of data (9 weeks with missing data were imputed using linear approximation). We group governorates by assigned outbreak clusters (separated by double solid lines) and include: the core outbreak (Sana'a, Sana'a City, Al-Hudaydah), immediate neighboring I (Amran, Al-Mahwit, Dhamar, Al-Bayda), immediate neighboring II (Raymah, Ibb, Taizz), northern (Hajjah, Al-Jawf, Sa'ada), southern (Al-Dhale'e, Abyan, Aden, Lahj), and eastern (Marib, Al-Maharah, Shabwah) clusters. We defined outbreak clusters according to similarities of outbreak signatures using smoothed rates and trivial derivative values calculated from KZ adaptive filters: $Z'_{t,s,3-5} = (Z^*_{t+1,s,3} + Z^*_{t+1,s,5})/2 - (Z^*_{t,s,3} + Z^*_{t,s,5})/2$. Data summarized in this table are, in part, graphically presented in Figure 3.

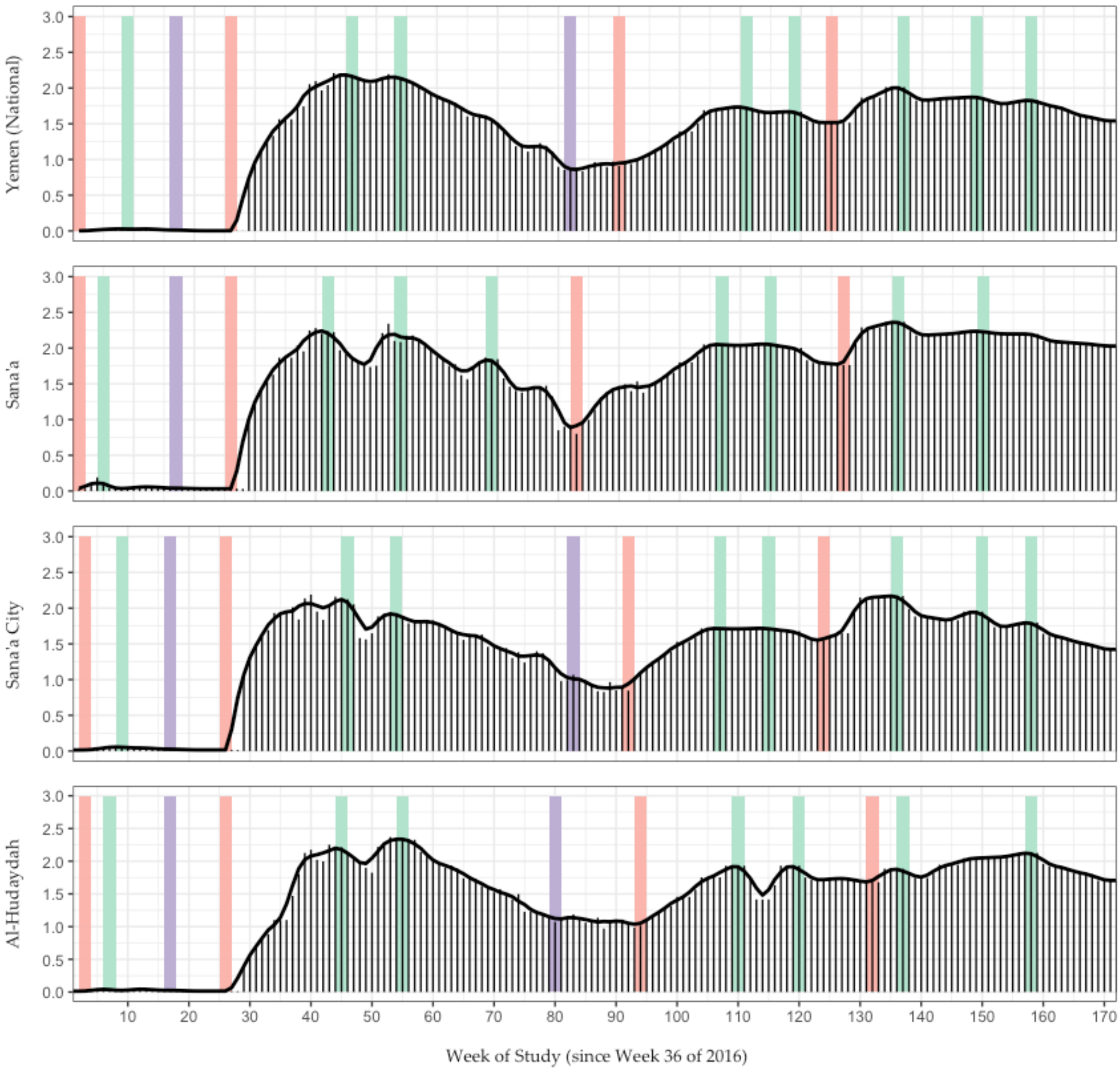

Supplementary Figure S1. A stacked multi-panel time series plot of the national Yemeni cholera outbreak signature and the governorates of Sana'a, Sana'a City, and Al-Hudaydah (core outbreak cluster). All plots show log10-transformed smoothed rates produced using Kolmogorov Zurbenko adaptive filters from Week 36 of 2016 (04-11 September) through Week 52 of 2019 (23-29 December). We estimated smoothed rates (laboratory-confirmed cholera cases per 100,000 persons) using the best performing smoother ($Z^*_{t,s,3-5}$) as the average of a 3-week ($Z^*_{t+1,s,3}$) and 5-week ($Z^*_{t+1,s,5}$) window size. We report the onset, peak, and resolution timing for each wave using red, green, and purple bars, respectively. Time series plots have a common horizontal axis of time reported in weeks since Week 36 of 2016 (defined as study week 0). We calculated all rates for the 172 weeks of time series data available in each governorate and nationally (we imputed 9 weeks of missing data using linear approximation). Data used to develop this visualization are reported in Tables 1 and 2 and Excel Table S3.

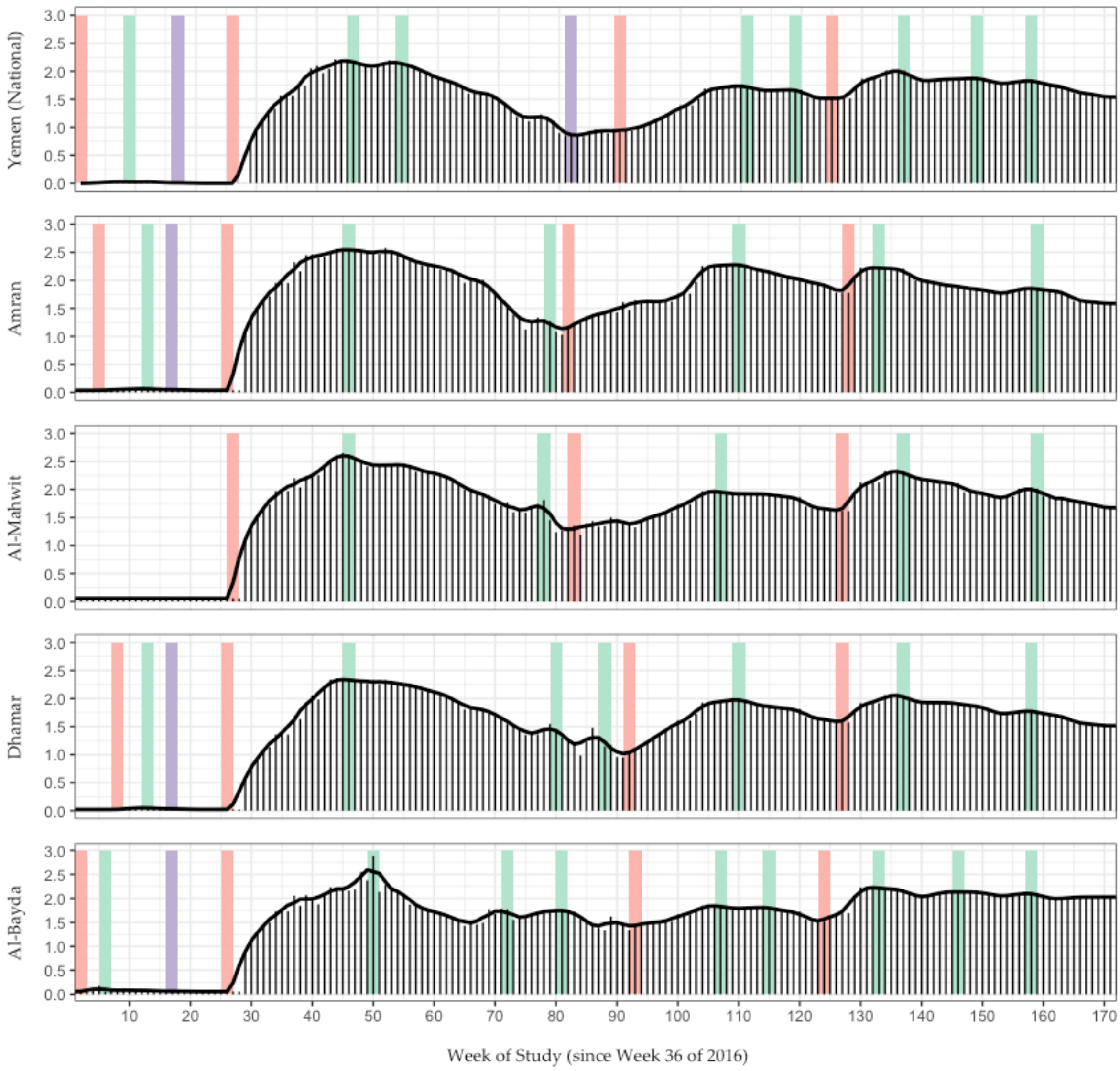

Supplementary Figure S2. A stacked multi-panel time series plot of the national Yemeni cholera outbreak signature and the governorates of Amran, Al-Mahwit, Dhamar, and Al-Bayda (immediate neighboring cluster I). All plots show log10-transformed smoothed rates produced using Kolmogorov Zurbenko adaptive filters from Week 36 of 2016 (04-11 September) through Week 52 of 2019 (23-29 December). We estimated smoothed rates (laboratory-confirmed cholera cases per 100,000 persons) using the best performing smoother ($Z^*_{t,s,3-5}$) as the average of a 3-week ($Z^*_{t+1,s,3}$) and 5-week ($Z^*_{t+1,s,5}$) window size. We report the onset, peak, and resolution timing for each wave using red, green, and purple bars, respectively. Time series plots have a common horizontal axis of time reported in weeks since Week 36 of 2016 (defined as study week 0). We calculated all rates for the 172 weeks of time series data available in each governorate and nationally (we imputed 9 weeks of missing data using linear approximation). Data used to develop this visualization are reported in Tables 1 and 2 and Excel Table S3.

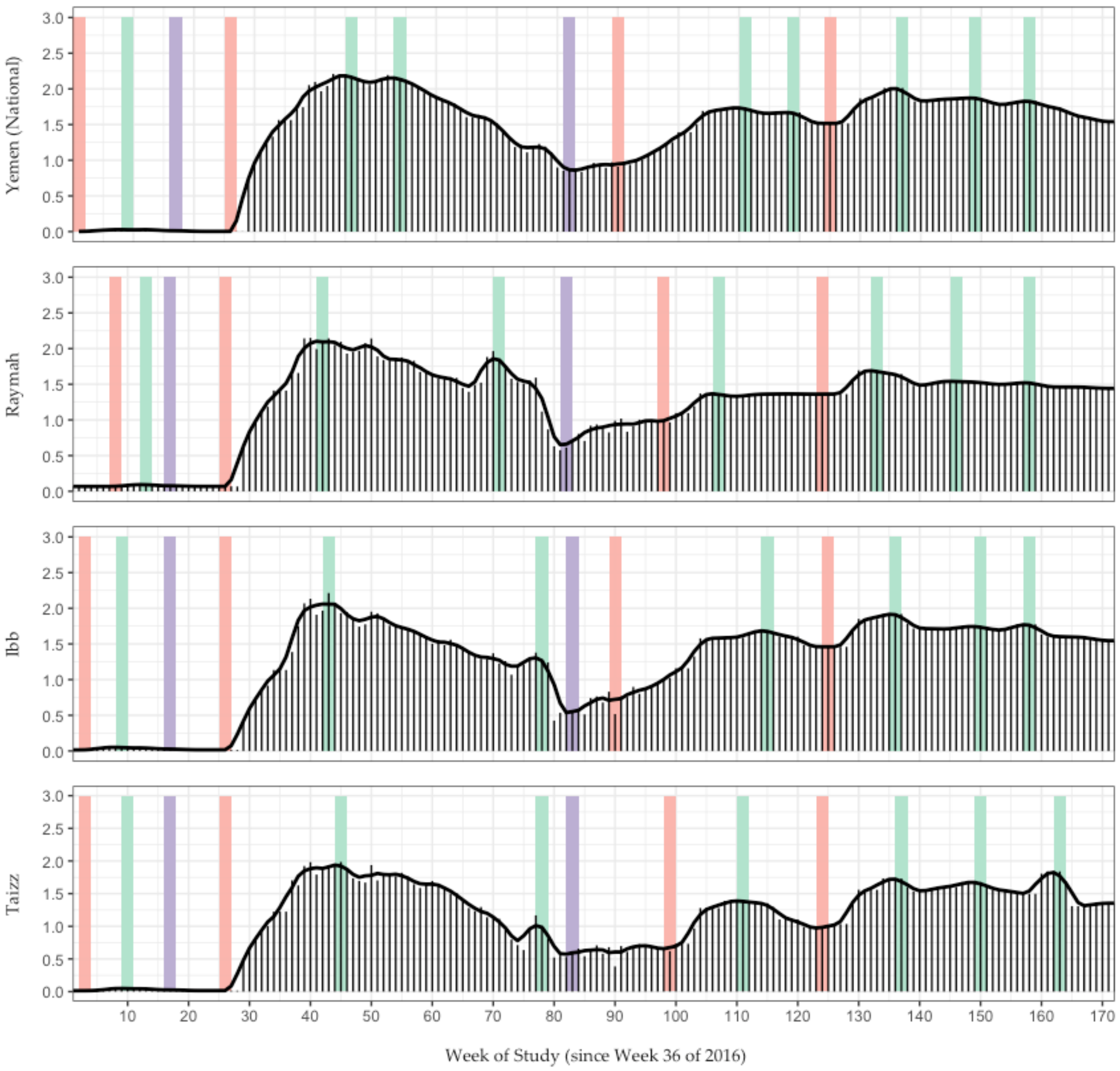

Supplementary Figure S3. A stacked multi-panel time series plot of the national Yemeni cholera outbreak signature and the governorates of Raymah, Ibb, and Taizz (immediate neighboring cluster II). All plots show log10-transformed smoothed rates produced using Kolmogorov Zurbenko adaptive filters from Week 36 of 2016 (04-11 September) through Week 52 of 2019 (23-29 December). We estimated smoothed rates (laboratory-confirmed cholera cases per 100,000 persons) using the best performing smoother ($Z^*_{t,s,3-5}$) as the average of a 3-week ($Z^*_{t+1,s,3}$) and 5-week ($Z^*_{t+1,s,5}$) window size. We report the onset, peak, and resolution timing for each wave using red, green, and purple bars, respectively. Time series plots have a common horizontal axis of time reported in weeks since Week 36 of 2016 (defined as study week 0). We calculated all rates for the 172 weeks of time series data available in each governorate and nationally (we imputed 9 weeks of missing data using linear approximation). Data used to develop this visualization are reported in Tables 1 and 2 and Excel Table S3.

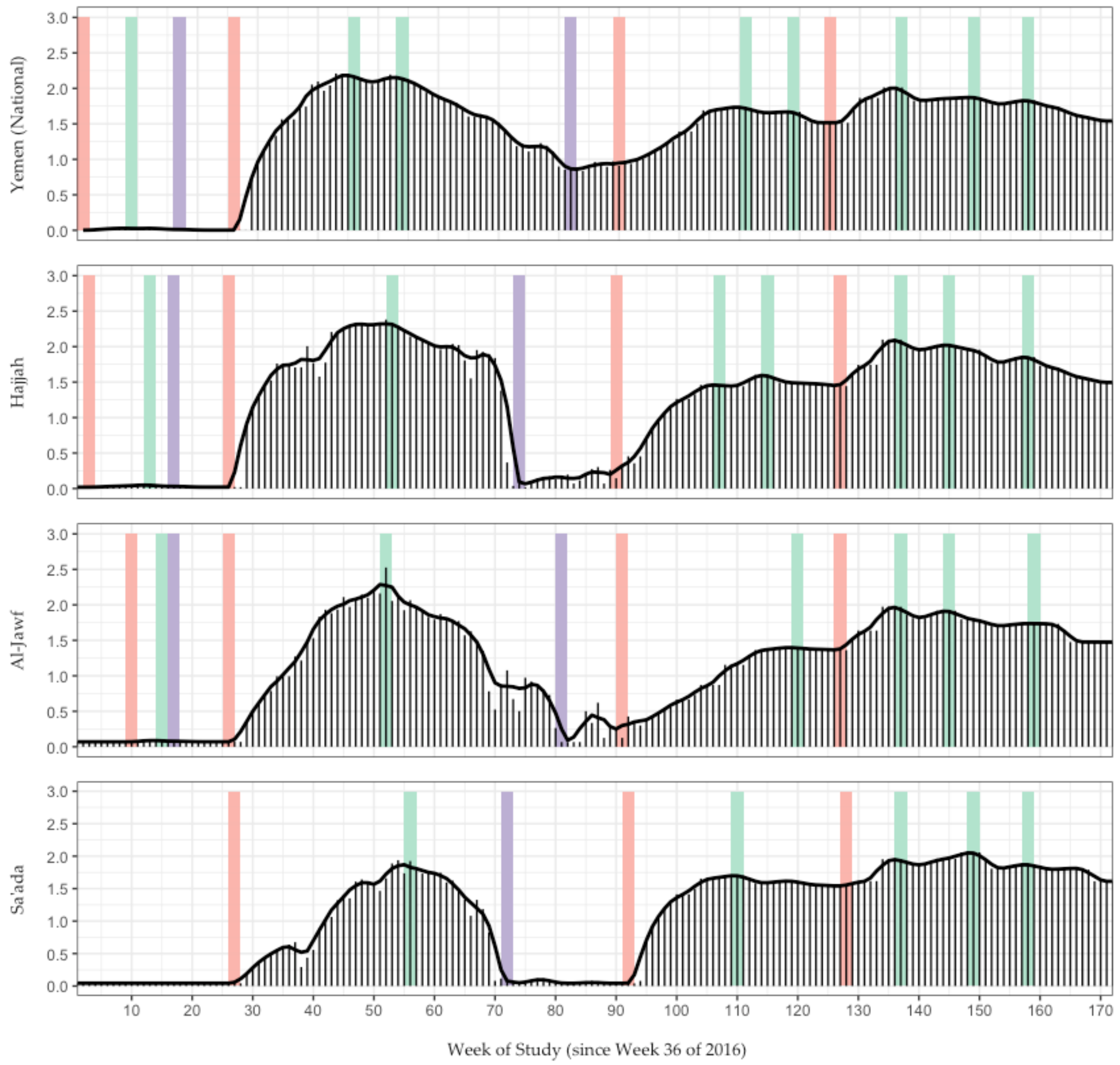

Supplementary Figure S4. A stacked multi-panel time series plot of the national Yemeni cholera outbreak signature and the governorates of Hajjah, Al-Jawf, and Sa'ada (remote northern cluster). All plots show log10-transformed smoothed rates produced using Kolmogorov Zurbenko adaptive filters from Week 36 of 2016 (04-11 September) through Week 52 of 2019 (23-29 December). We estimated smoothed rates (laboratory-confirmed cholera cases per 100,000 persons) using the best performing smoother ($Z^{*}_{t,s,3-5}$) as the average of a 3-week ($Z^{*}_{t+1,s,3}$) and 5-week ($Z^{*}_{t+1,s,5}$) window size. We report the onset, peak, and resolution timing for each wave using red, green, and purple bars, respectively. Time series plots have a common horizontal axis of time reported in weeks since Week 36 of 2016 (defined as study week 0). We calculated all rates for the 172 weeks of time series data available in each governorate and nationally (we imputed 9 weeks of missing data using linear approximation). Data used to develop this visualization are reported in Tables 1 and 2 and Excel Table S3.

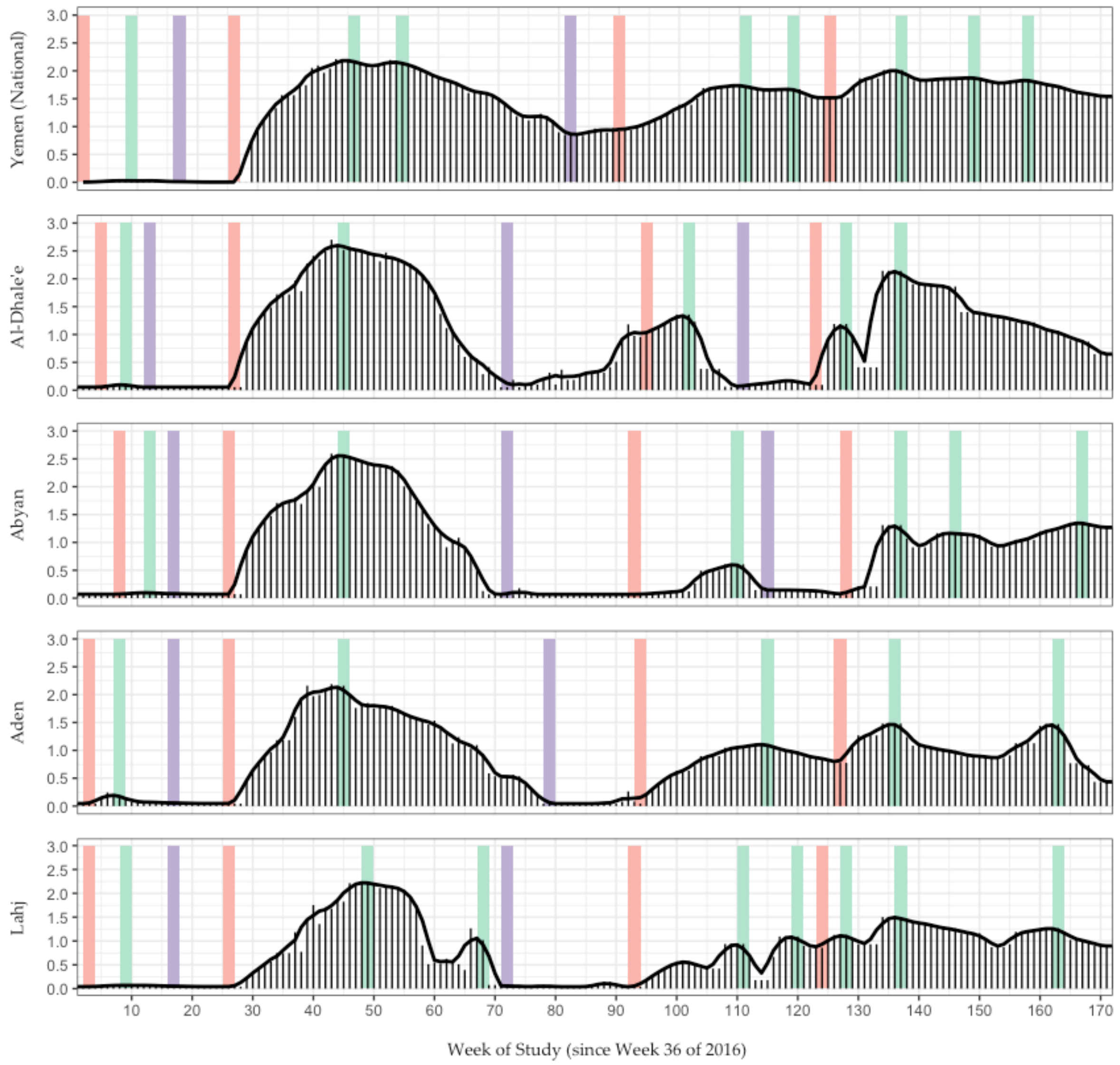

Supplementary Figure S5. A stacked multi-panel time series plot of the national Yemeni cholera outbreak signature and the governorates of Al-Dhale'e, Abyan, Aden, and Lahj (remote southern cluster). All plots show log10-transformed smoothed rates produced using Kolmogorov Zurbenko adaptive filters from Week 36 of 2016 (04-11 September) through Week 52 of 2019 (23-29 December). We estimated smoothed rates (laboratory-confirmed cholera cases per 100,000 persons) using the best performing smoother ($Z^*_{t,s,3-5}$) as the average of a 3-week ($Z^*_{t+1,s,3}$) and 5-week ($Z^*_{t+1,s,5}$) window size. We report the onset, peak, and resolution timing for each wave using red, green, and purple bars, respectively. Time series plots have a common horizontal axis of time reported in weeks since Week 36 of 2016 (defined as study week 0). We calculated all rates for the 172 weeks of time series data available in each governorate and nationally (we imputed 9 weeks of missing data using linear approximation). Data used to develop this visualization are reported in Tables 1 and 2 and Excel Table S3.

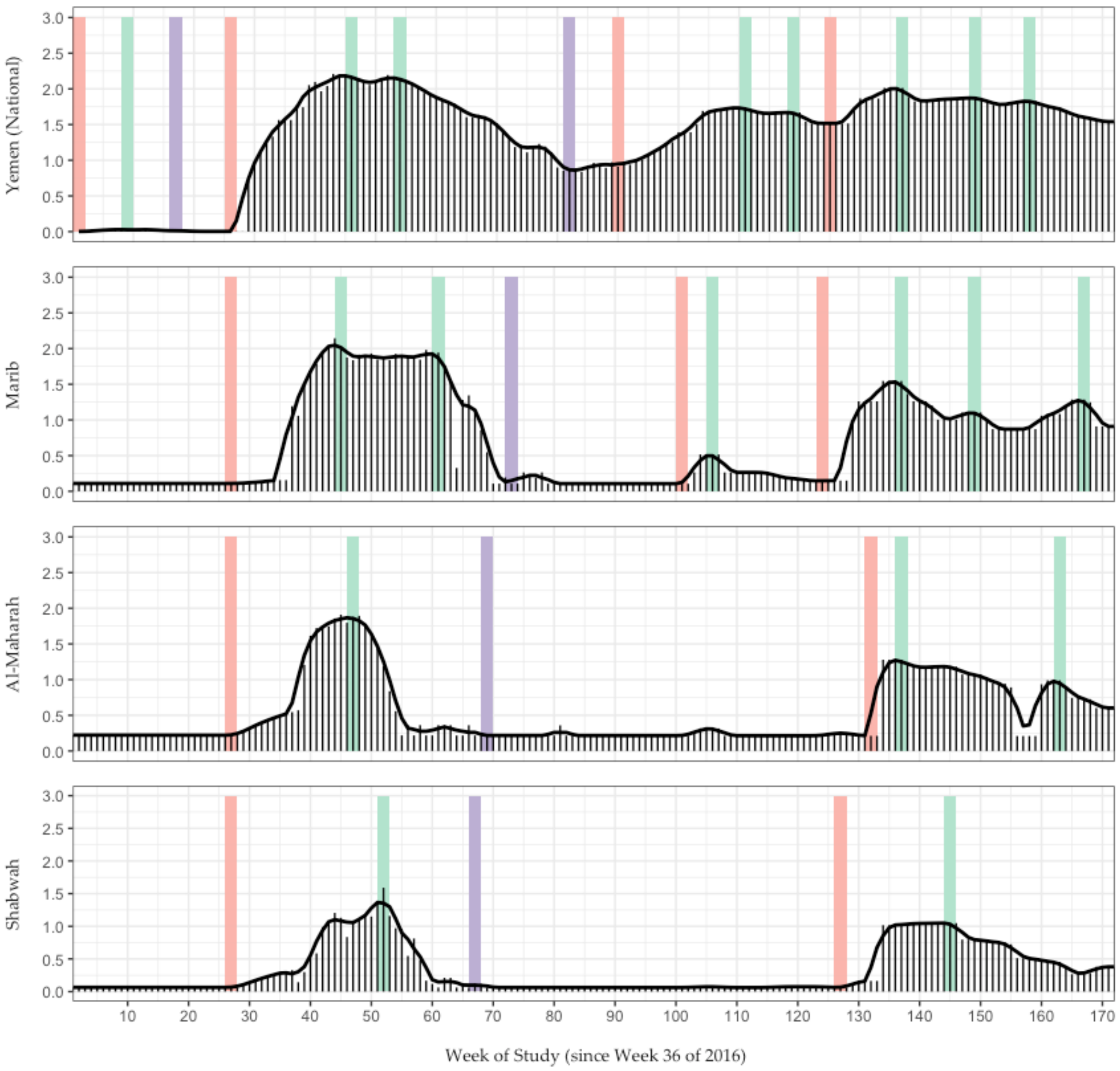

Supplementary Figure S6. A stacked multi-panel time series plot of the national Yemeni cholera outbreak signature and the governorates of Marib, Al-Maharah, and Shabwah (remote eastern cluster). All plots show log10-transformed smoothed rates produced using Kolmogorov Zurbenko adaptive filters from Week 36 of 2016 (04-11 September) through Week 52 of 2019 (23-29 December). We estimated smoothed rates (laboratory-confirmed cholera cases per 100,000 persons) using the best performing smoother ($Z^*_{t,s,3-5}$) as the average of a 3-week ($Z^*_{t+1,s,3}$) and 5-week ($Z^*_{t+1,s,5}$) window size. We report the onset, peak, and resolution timing for each wave using red, green, and purple bars, respectively. Time series plots have a common horizontal axis of time reported in weeks since Week 36 of 2016 (defined as study week 0). We calculated all rates for the 172 weeks of time series data available in each governorate and nationally (we imputed 9 weeks of missing data using linear approximation). Data used to develop this visualization are reported in Tables 1 and 2 and Excel Table S3.
